# Adherence to phase I cardiac rehabilitation in post-PCI patients: a latent class analysis

**DOI:** 10.3389/fcvm.2025.1460855

**Published:** 2025-02-24

**Authors:** Zhuo Chai, Yuxuan Fan, Xue Gong, Yue Zhang, Yanling Hu, Xiaowei Li, Zhiqing Fan, Yongkui Han

**Affiliations:** ^1^ Department of Nursing, Harbin Medical University, Daqing, Heilongjiang, China; ^2^ Department of Rehabilitation, Tongji Hospital Affiliated to Tongji University, Tongji University School of Medicine, Shanghai, China; ^3^ Department of Cardiology, Daqing Oilfield General Hospital, Daqing, Heilongjiang, China

**Keywords:** cardiac rehabilitation, percutaneous coronary intervention, adherence, latent class analysis, China

## Abstract

**Background:**

Coronary heart disease seriously jeopardizes human health and has become a principal public health problem of global concern. While percutaneous coronary intervention (PCI) repairs narrowed arteries and extends patients' lives, cardiac rehabilitation offers additional benefits post-PCI. Numerous previous studies have shown that cardiac rehabilitation can inhibit the progression of atherosclerotic plaques in patients after coronary intervention, effectively controlling patients' clinical symptoms and improving their quality of life. However, the current status of adherence to phase I cardiac rehabilitation is poor, and the variation in adherence to phase I cardiac rehabilitation among post-PCI patients in China are not well understood. This study aimed to identify the heterogeneity of adherence to phase I cardiac rehabilitation and its influencing factors in post-PCI patients through latent class analysis (LCA) to support individualized interventions.

**Methods:**

PCI patients (*N* = 212) admitted to the Cardiac Rehabilitation Center of Daqing Oilfield General Hospital in Heilongjiang Province were selected to complete the General Demographic Information Questionnaire, Cardiac Rehabilitation Adherence Scale for Coronary Heart Disease Patients, Cardiac Rehabilitation Knowledge Questionnaire, Patient Health Questionnaire-9 (PHQ-9), Social Support Rating Scale, Tampa Scale for Kinesiophobia-Swedish Version (TSK-SV), and Chronic Disease Resource Survey Questionnaire at the end of phase I cardiac rehabilitation. Latent class analysis identified potential categories of adherence to phase I cardiac rehabilitation in post-PCI patients. Logistic regression analyzed the factors influencing the different categories.

**Results:**

Adherence to phase I cardiac rehabilitation in post-PCI patients was classified into three groups: Good Adherence (31.2%), Poor Nutritional and Psychological Management (32.0%), and Lack of Exercise (36.8%). Limited social support, poor utilization of chronic disease resources, low education level, a history of alcohol consumption, and kinesiophobia are factors influencing the different latent subgroups (*P* < 0.05).

**Conclusion:**

Heterogeneity exists in the adherence to phase I cardiac rehabilitation of post-PCI patients. Healthcare professionals should implement targeted interventions based on the characteristics of each category to improve adherence.

## 1 Introduction

### 1.1 Background

Cardiovascular disease (CVD) is the leading cause of death, accounting for approximately one-third of all deaths (1). The control and prevention of chronic non-communicable diseases, including cardiovascular diseases, are a major focus in the medical field (2). Currently, relevant disease reports indicate that there are a large number of high-risk groups for CVD in China, and it is estimated that the number of current CVD patients reaches 330 million (3). However, with the continuous improvement of medical care, more and more CVD patients are receiving Percutaneous Coronary Intervention (PCI) treatment. PCI can rapidly restore blood circulation in coronary arteries, improve myocardial blood supply, and prevent myocyte necrosis. These effects significantly improve the survival rate of CVD patients and reduce CVD-related disability and death rates (4, 5). The recovery period after PCI in patients with CVD is critical and necessitates the implementation of a comprehensive cardiac rehabilitation strategy to reduce the risk of recurrent events and improve overall quality of life.

Cardiac rehabilitation is a multidisciplinary effort led by the cardiovascular team to minimize the physiological and psychological impact of CVD on patients, lower the risk of sudden death or reinfarction, and deliver long-term, comprehensive medical care to improve patients' quality of life (6). Currently, cardiac rehabilitation is recognized both domestically and internationally as being divided into 3 phases, i.e., Phase I (in-hospital rehabilitation), Phase II (out-of-hospital early rehabilitation or outpatient rehabilitation), and Phase III (out-of-hospital long-term rehabilitation) (7). Phase I cardiac rehabilitation is a critical period for establishing the concept of patient rehabilitation, promoting the recovery of cardiac function, and conducting rehabilitation education. It is an area that requires immediate standardization and regulatory oversight. However, globally, cardiac rehabilitation for post-PCI patients is often underutilized (8, 9), with dropout rates ranging from 25% to 42% in the U.S (10, 11), 56% in New Zealand (12), 28% to 45% in Canada (13), and 55% to 82% in Iran (14, 15).

The adherence to cardiac rehabilitation among patients after PCI is influenced by various factors. A meta-analysis showed that demographic factors such as age, gender, and economic status, as well as disease-related factors, all have a certain degree of impact on the rehabilitation adherence of PCI patients (16). Additionally, lower education level, living alone, being divorced or single, and poorer social support are associated with lower adherence (17). Behaviors such as smoking, depression (18), and alcohol consumption (19) also affect the attendance rate of patients in cardiac rehabilitation. The American Heart Association (AHA) believes that a lack of awareness of the benefits of cardiac rehabilitation is the main reason for its underutilization (20). A cross-sectional study by Keessen et al., surveying 152 patients participating in cardiac rehabilitation, found that 45.4% of patients had a high level of exercise-related fear (21). The study also found that greater utilization of chronic disease resources increases patients' likelihood of participating in cardiac rehabilitation (22). In summary, adherence to cardiac rehabilitation is influenced by multiple dimensions, including sociodemographic factors, disease-related factors, and psychosocial factors. However, the results from different studies are not entirely consistent, and there is currently a lack of a comprehensive and systematic theoretical framework to study these influencing factors.

The development of cardiac rehabilitation in China was pioneered by Prof. Hu Daiyi, who proposed the “five prescriptions” for cardiac rehabilitation: the drug prescription, the nutritional prescription, the exercise prescription (form, intensity, frequency, and duration of exercise), the psychological prescription, and the smoking cessation prescription (23, 24). Existing studies on adherence to cardiac rehabilitation have focused more on adherence to rehabilitation exercise (25) and medication administration (26), and less on other aspects of the rehabilitation prescription. Thus, the evaluation of adherence to phase I cardiac rehabilitation lacks comprehensiveness. Wen Xiaohui and others (27) constructed a cardiac rehabilitation adherence evaluation scale for patients with coronary artery disease based on the Delphi method, including adherence evaluations for exercise prescription, medication prescription, nutritional prescription, risk factor management, smoking and alcohol cessation prescription, and psychological prescription (including sleep management), making it more systematic and comprehensive. Therefore, this study employed this scale to evaluate phase I cardiac rehabilitation among post-PCI patients.

In addition, most studies on phase I cardiac rehabilitation for post-PCI patients treat patients as a homogeneous group, ignoring heterogeneity at the individual level, limiting the guidance of specific clinical practice. Latent class analysis (LCA) is a valuable methodological tool for delineating subtypes of adherence patterns in phase I cardiac rehabilitation among post-PCI patients. LCA describes population heterogeneity in terms of individual differences in a set of behaviors or characteristic statistical methods, an approach known as a person-centered perspective (28). Owing to these advantages, it has been used in many fields, such as medicine, sociology, and psychology (29, 30), to identify unobserved subgroups, especially potential adherence types (31, 32). Using LCA, researchers can categorize post-PCI patients in phase I cardiac rehabilitation into distinct subgroups and make each subgroup representative of adherence for people with similar characteristics. Moreover, incorporating covariates enables researchers to gain a more intricate understanding of the determinants influencing adherence to cardiac rehabilitation. To our knowledge, no LCA studies have been conducted to examine patterns of adherence to cardiac rehabilitation among PCI patients.

This study aims to explore adherence to cardiac rehabilitation in patients after PCI using a cross-sectional design and Latent Class Analysis (LCA), with a focus on identifying different subtypes of adherence. Variance analysis and logistic regression will be used to assess the underlying factors influencing these subtypes. The study is expected to fill the existing gap in research on post-PCI cardiac rehabilitation adherence and provide more precise evidence for future clinical practice, ultimately helping to optimize Phase I cardiac rehabilitation services in hospital settings.

### 1.2 Theoretical framework

The Health Ecological Model (HEM) applies ecological theory to human health, emphasizing that health results from interactions between individuals and their environment. The model has a five-tier structure, starting with a core layer of personal traits and descending through behavioral characteristics, interpersonal networks, work and living conditions, and the policy environment. The factors included in the five-layer structure interact with each other and jointly influence human health (33). In this study, we systematically investigated the factors influencing adherence to cardiac rehabilitation in post-PCI patients. We explored the literature by reviewing domestic and international sources and selected representative factors from each level of the Health Ecological Model. The general idea of the model is as follows: The personal traits layer incorporates demographic information (age, gender) and disease-related information (number of previous medical conditions); the behavioral traits layer incorporates smoking history, alcohol consumption history, depressed mood, knowledge of cardiac rehabilitation, kinesiophobia, and use of resources for chronic diseases; the interpersonal network layer incorporates social support, residency, and marital status; the work and living conditions layer incorporates literacy level and per capita monthly household income; and the policy environment layer incorporates types of healthcare payment systems.

## 2 Methods

### 2.1 Sample

The study recruited PCI patients admitted to the Cardiac Rehabilitation Center of Daqing Oilfield General Hospital in Heilongjiang Province between October 2023 and April 2024. The inclusion and exclusion criteria for study participants are detailed in Table 1.

**Table 1:** Inclusion and exclusion criteria for study participants.

Criteria	Details
Inclusion criteria	1. First-time PCI with successful treatment (no postoperative complications);
	2. Meeting the indications for cardiac rehabilitation, including: (1) no new or recurrent chest pain, significant arrhythmias within the past 8 h; (2) no elevation in creatine kinase or troponin levels, and no new signs of heart failure decompensation; (3) resting heart rate between 50 and 120 beats per minute, resting systolic blood pressure (SBP) 90–150 mmHg, and resting diastolic blood pressure (DBP) 60–100 mmHg; (4) oxygen saturation >90% or recent decrease <4%;
	3. Age ≥18 years;
	4. Ability to hear, read, and write in Chinese, and independently complete the questionnaire during the survey;
	5. Voluntary participation in the study and signed informed consent
Exclusion Criteria	1. Unstable conditions, including but not limited to multi-organ failure [e.g., acute heart failure (NYHA class III–IV), acute kidney failure], severe hypotension (systolic blood pressure <90 mmHg), significant postoperative bleeding, or any other critical condition that may impact safety or compliance with the rehabilitation program;
	2. Not meeting cardiac rehabilitation indications: including malignant arrhythmias (e.g., sustained ventricular fibrillation), uncontrolled hypertension (systolic blood pressure ≥180 mmHg or diastolic blood pressure ≥110 mmHg), severe respiratory dysfunction, etc.;
	3. Cognitive impairment or psychiatric disorders (e.g., moderate to severe Alzheimer's disease, major depressive disorder, etc.) that prevent cooperation in the study;
	4. Unwillingness to participate in the study or refusal to sign the informed consent form

For Latent Class Analysis (LCA), when using Bayesian Information Criterion (BIC) and Entropy as the primary indicators for model selection, a sample size of *N* > 200 participants is recommended (34). In this study, the final sample size of 212 participants ensured sufficient statistical power to identify distinct latent classes while maintaining model stability and interpretability. Furthermore, our sample size fully meets the recommended range for multifactor analysis, thereby supporting the robustness of the study results.

### 2.2 Measures

#### 2.2.1 General demographic information questionnaire

It was designed by the researchers themselves based on an extensive review of relevant literature and after discussion at an expert panel meeting. The questionnaire includes gender, age, education level, per capita monthly household income, mode of payment for medical expenses, smoking history, alcohol consumption history, and the number of previous medical conditions.

#### 2.2.2 The cardiac rehabilitation adherence evaluation scale for coronary heart disease patients

The Cardiac Rehabilitation Adherence Evaluation Scale for Coronary Heart Disease Patients, developed by Wen Xiaohui et al., was selected (27). It includes exercise adherence, medication adherence, risk factor management, smoking and alcohol cessation adherence, nutritional management adherence, and psychological management adherence. “Yes” answers score 1 point, while “No” answers score 0 points. The total score range is 0–33 points; the higher the score, the better the adherence to cardiac rehabilitation in patients with coronary heart disease. For positive questions, “Yes” scores 1 point and “No” scores 0 points. For negative questions, the scoring is reversed. In the medication adherence section, questions 3, 4, and 5 were reverse-scored. Adherence was categorized as high (33–25 points) and low (24–0 points) according to the criteria. The Cronbach's alpha coefficient for this scale in this study was 0.807, indicating reliable internal consistency. The detailed contents of the scale are provided in Supplementary Table 1. Permission to use the scale was obtained from the original authors.

#### 2.2.3 Cardiac rehabilitation knowledge questionnaire

This questionnaire was compiled by Qian Zhang (35) according to the Chinese Expert Consensus on Coronary Heart Disease Cardiac Rehabilitation/Secondary Prevention. The content includes four aspects: basic knowledge of cardiac rehabilitation, knowledge related to exercise, knowledge related to risk factors, and health responsibility. Basic knowledge of cardiac rehabilitation, exercise-related knowledge, and risk factor-related knowledge each have two options: “know” and “don't know.” “Know” scores 1 point, and “don't know” scores 0 points. Health responsibility has three options: “very necessary,” “necessary,” and “unnecessary,” scoring 3, 2, and 1 points, respectively. The total score is 48, with a higher score indicating better understanding and knowledge of cardiac rehabilitation. The Cronbach's alpha coefficient for this scale in this study was 0.745, indicating reliable internal consistency.

#### 2.2.4 The patient health questionnaire 9 (PHQ-9)

This is part of the PHQ scale, a self-rating depression scale with 9 items (36). The items are rated on a 4-point scale: No depression: 0–4; Mild depression: 5–9; Moderate depression: 10–14; Severe depression: 15–17. The Cronbach's alpha coefficient for this scale in this study was 0.725, indicating reliable internal consistency.

#### 2.2.5 Social support rating scale

The Social Support Rating Scale, compiled by Xiao Shuiyuan (37) based on foreign data, was used. The scale has 10 items, including objective support (items 2, 6, and 7), subjective support (items 1, 3, 4, and 5), and utilization of support (items 8, 9, and 10). A higher total score indicates a higher level of social support. The Cronbach's alpha coefficient for this scale in this study was 0.761, suggesting reliable internal consistency.

#### 2.2.6 The Tampa scale for kinesiophobia heart (TSK-SV heart)

It was compiled by Bäck (38) and includes 4 dimensions: fear of injury, decline in one's functioning, avoidance of exercise, and perceived danger to the heart, with a total of 17 items scored on a 4-point Likert scale, where 1 indicates strong disagreement, 2 indicates disagreement, 3 indicates agreement, and 4 indicates strong agreement. The higher the score, the stronger the kinesiophobia. A score of ≥37 indicates a clinically significant level of kinesiophobia. The Cronbach's alpha coefficient for this scale in this study was 0.878, indicating reliable internal consistency.

#### 2.2.7 Chronic disease resource survey questionnaire

It was compiled by Glasgow et al. (33) in 2000 based on the social-ecological theory model, translated and introduced into China by Huiqin Zhong et al. (39). It was used to evaluate the support of multiple social resources for chronic disease management. The scale consists of 21 items across 7 dimensions: healthcare professionals, family and friends, individuals, neighborhoods or communities, media and policies, organizations, and workplaces. The total score ranges from 21 to 105, with higher scores indicating greater utilization of resources for chronic disease management. The Cronbach's alpha coefficient for this scale in this study was 0.891, suggesting reliable internal consistency.

### 2.3 Phase I cardiac rehabilitation content

This study's Phase I cardiac rehabilitation is strictly implemented according to the “Chinese Expert Consensus on Coronary Heart Disease Rehabilitation and Secondary Prevention” (40), and personalized adjustments are made based on the patient's individual condition. The duration of Phase I cardiac rehabilitation can be shortened or extended based on the patient's actual situation. For example, the rehabilitation period may be shortened for patients who recover more quickly, with the main rehabilitation goals potentially being completed 3–5 days post-surgery, allowing for discharge. For patients with more severe comorbidities or slower recovery, the rehabilitation period may be extended to 7 days or longer. In this study, the average hospital stay for patients post-PCI was 5.5 days. All patients were undergoing PCI for the first time, so none had prior experience with cardiac rehabilitation. At discharge, all patients completed assessments of their adherence to rehabilitation and evaluations of various influencing factors. The specific rehabilitation procedures and assessment details are provided in Table 2.

**Table 2:** Cardiac rehabilitation content post-PCI (1–7 days).

Time	Day 1	Day 2	Day 3	Day 4	Day 5	Day 6–7
Energy consumption	1–2 METs	2–3 METs	1–2 METs	2–3 METs	3–4 METs	4–5 METs
Frequency	Once per hour	Twice daily, with 3–4 h intervals	Twice daily	Twice daily	Twice daily	Twice daily
Intensity	RPE maintained at 6–8 (Very Light)	RPE maintained at 7–9 (Light)	RPE maintained at 9–11 (Somewhat Hard)	RPE maintained at 9–11 (Somewhat Hard)	RPE maintained at 11–13 (Hard)	RPE maintained at 11–13 (Hard)
Type	Passive joint movements, bed exercises	Bedside activities, active/passive joint movements	Warm-up exercise, slow walking (15–25 m)	Indoor activity and stretching exercises, moderate walking (25–50 m)	Walking (100–150 m) or cycling (20–40 W), stair climbing	Walking (200–400 m), cycling, stair climbing
Time	5–10 min per session, 8–10 sessions throughout the day	10–15 min per session	10–15 min per session	15–20 min per session	20–30 min per session	30 min per session
Monitoring methods	RPE and heart rate monitored to ensure safe exertion levels	RPE, heart rate monitored, ensuring heart rate increases by no more than 10–15 bpm above baseline	RPE, heart rate checked. Heart rate should remain within the target range (+10–15 bpm)	RPE and heart rate monitoring. Ensuring heart rate stays within the prescribed increase	RPE, heart rate controlled, target heart rate within the prescribed zone (+20 bpm)	RPE, heart rate, 6 MWT performed to assess functional progress. Heart rate maintained within the target range (+20 bpm)
Daily life	Absolute bed rest, eating with help from caregivers	Bed rest, eating in bed, with assistance in washing face, bathing, and dressing	Mostly independent, can sit in a chair, sit in a wheelchair to the ward and treatment rooms	Fully independent in daily activities, allowed to get out of bed under supervision, walk to the bathroom, ward, and treatment rooms	Fully independent, walking to reception room/phone room, able to walk in the ward corridor anytime	Continue activities mentioned earlier, with slightly increased intensity
Education and promotion	Introduce Coronary Care Unit (CCU), emotional regulation; dietary advice: drink 1–2 L of water within 6–8 h, urine output must reach 800 ml to help the excretion of contrast agent through kidneys	Introduce the rehabilitation team and program, quit smoking, provide educational materials	Introduce heart anatomy and the pathophysiology of coronary heart disease	Educate on risk factors for coronary heart disease and their control	Educate on medications, diet, exercise, heart rate monitoring, and sexual activity	Educate on follow-up items, psychological counseling, and precautions
Other considerations	Management of emergency situations; management of puncture wound	Rest for 15–30 min after each activity	Rest for 15–30 min after each activity	All activities should be performed within tolerance	All activities should be performed within tolerance	Prepare for discharge, conduct evaluations including the coronary artery disease patient compliance evaluation scale, heart rehabilitation knowledge questionnaire, PHQ-9, social support assessment scale, heart phobia scale, and chronic disease resource utilization survey

(1) The program should be implemented on an individualized basis, with the next step in the rehabilitation process determined by the patient's progress at each stage. The duration of each stage may be shortened or extended as necessary. (2) Rehabilitation must be carried out under continuous electrocardiographic monitoring, with close observation of cardiovascular parameters. (3) Starting from day 3 of the program, the walking distance is applicable to patients who underwent radial artery access. For patients who underwent femoral artery access, upper limb exercises should be substituted, as significant lower limb movement should be avoided within the first week. (4) Indicators for halting activities: the activity should be immediately stopped in the presence of any of the following conditions, and the rehabilitation program should be extended as appropriate based on the situation: (a) heart rate ≥110 beats/min; (b) chest pain, chest tightness, shortness of breath, palpitations, dizziness, fainting, pale complexion, or profuse sweating; (c) ST segment depression ≥0.1 mV or elevation ≥0.2 mV during activity; (d) systolic blood pressure increases by 20 mmHg (1 mmHg = 0.133 kPa) or more, or systolic blood pressure fails to rise and instead decreases; (e) severe arrhythmias; (f) exercise tests can be performed as early as 1–2 weeks post-PCI, but this should be decided by the clinician based on each individual patient's condition. (5) RPE: a subjective method for assessing exercise intensity based on the patient's own perception of effort. The Borg RPE scale is commonly used, with a range from 6 to 20, where higher numbers represent higher exercise intensity. (6) 6 MWT, six-minute walk test.

In this study, patients undergoing PCI had an average hospital stay of 5.5 days. From day 1 to day 5 post-PCI, patients received regular clinical evaluations. At discharge, compliance and other influencing factor scales were assessed. The specific rehabilitation program and daily monitoring methods based on the FITT principle (Frequency, Intensity, Type, and Time) are outlined in Table 2.

### 2.4 Data collection

**Figure 1:**
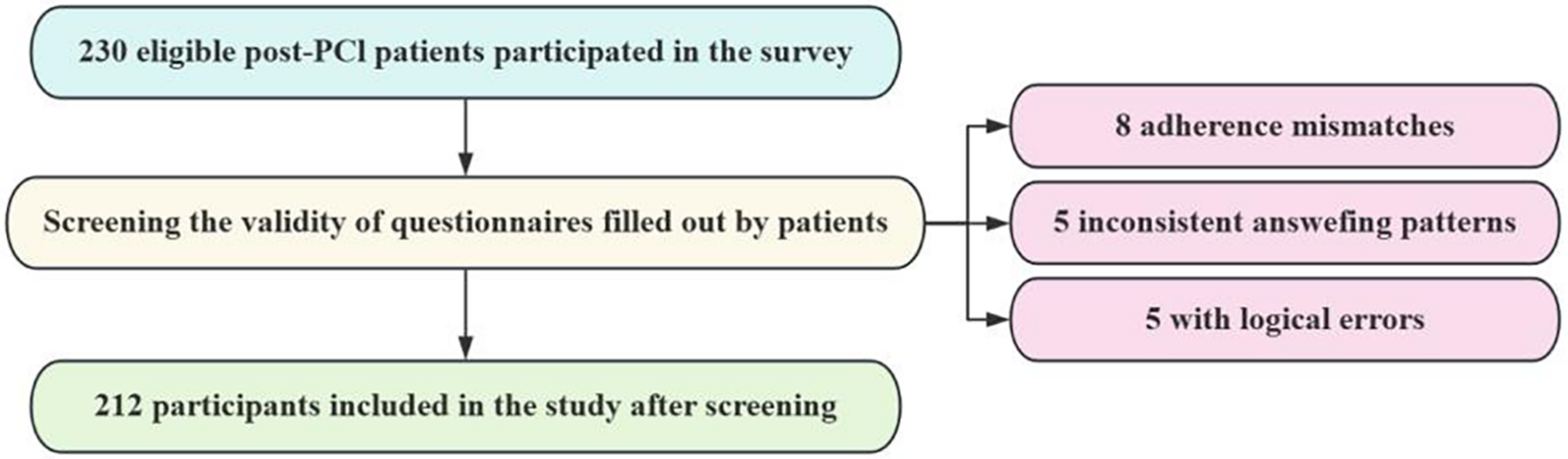
Participant selection flowchart.

The survey was conducted from October 2023 to April 2024 at Daqing Oilfield General Hospital. After obtaining consent from the data collection unit and the patients, researchers administered questionnaires to patients who met the inclusion and exclusion criteria using a standardized script. General demographic data were collected upon patient admission, while the Coronary Heart Disease Patient Cardiac Rehabilitation Adherence Evaluation Scale, Cardiac Rehabilitation Knowledge Questionnaire, PHQ-9, Social Support Rating Scale, TSK-SV Heart, and Chronic Disease Resource Utilization Survey Questionnaire were administered after the patient's condition had stabilized post-PCI and at the completion of Phase I cardiac rehabilitation. The completed questionnaires were collected on-site, checked immediately, and corrected for any obvious errors or omissions by the patients before submission. A total of 230 questionnaires were distributed. Of these, 5 patients showed inconsistent answering patterns, 5 had questionnaires with obvious logical errors, and 8 patients' self-reported adherence to Phase I cardiac rehabilitation did not align with their actual adherence. After excluding these responses, 212 valid questionnaires were collected, resulting in an effective response rate of 92.2%. Details of the participant selection process are illustrated in Figure 1.

### 2.5 Statistical analysis

Data were double-entered using SPSS 27.0, and cardiac rehabilitation adherence was analyzed for potential categories using Mplus 8.0. General information was analyzed using descriptive analysis methods, and categorical variables were expressed as the number of cases and percentage. A Latent Class Model (LCM) was constructed using Mplus 8.0 software, with the following evaluation indices: the Akaike Information Criterion (AIC), the Bayesian Information Criterion (BIC), adjusted BIC (aBIC), and entropy to determine the accuracy of the classification. The smaller the AIC, BIC, and aBIC, the better the model fit. The entropy value ranges from 0 to 1, with values closer to 1 indicating better fit. The Likelihood Ratio Test (Lo-Mendell-Rubin, LMR) and the Bootstrap-based likelihood ratio test were used to compare the fit of two neighboring models. A *P*-value of <0.05 indicated that the k-model fit better than the k-1 model. After determining the best latent profile model and defining the classifications, statistical analysis was performed using SPSS 27.0. Normally distributed continuous data were expressed as mean ± standard deviation, and categorical data were expressed as frequency and percentage (%). Correlations between the independent variables and adherence in different subgroups were assessed using the Chi-square test or one-way ANOVA. Statistically significant variables in the univariate analysis were included in a stepwise multivariate logistic regression analysis. Odds ratios (*ORs*) and corresponding 95% confidence intervals (95% *CIs*) were calculated to assess the results of the regression analysis. The significance level was set at *α* = 0.05, with *P* < 0.05 considered statistically significant.

## 3 Results

### 3.1 Sample characteristics

A total of 212 questionnaires were collected in this study, with the majority of participants being middle-aged and older adult, and predominant number of males (68.9%). Of the participants, 196 were married (92.5%), 9.0% lived alone, and 98.6% had health insurance. The per capita monthly household income of the participants was greater than 5,000 yuan (49.1%). Additional information is presented in Table 3.

**Table 3:** General demographic data and clinical characteristics of patients after PCI (*N* = 212).

Item	Classification	Number (%)	Item	Classification	Number (%)
Gender	Male	146 (68.9)	Education level	Primary and below	116 (28.1)
	Female	66 (31.1)		Junior	139 (33.7)
Age	<50	24 (11.3)		High school or junior college	108 (26.2)
	50–60	62 (29.2)		University and above	50 (12.1)
	60–70	78 (36.8)	Monthly per capita household income	<1,000 yuan	3 (1.4)
	>70	48 (22.6)		1,000–3,000 yuan	34 (16.0)
Residence	Living alone	19 (9.0)		3,001–5,000 yuan	71 (33.5)
	Not living alone	193 (91.0)		>5,000 yuan	104 (49.1)
Marital status	Married	196 (92.5)	Number of past histories	0	59 (27.8)
	Other	16 (7.5)		1	100 (47.2)
Methods of payment of medical expenses	Self-financed	3 (1.4)		2	38 (17.9)
	Medical insurance	209 (98.6)		3	15 (7.1)
Smoking history	Yes	105 (49.5)	Drinking history	Yes	120 (56.6)
	No	107 (50.5)		No	92 (43.4)

### 3.2 Latent profile analysis

A total of four potential category models were fitted in this study, and the fitting metrics for each model are shown in Table 4. The results indicated that Model 3 had the smallest values for AIC, BIC, and aBIC; the *P*-values for LMR and BLRT were <0.001 for Model 3; and the Entropy value of 0.961 was also close to 1. Therefore, after comprehensively comparing all the fitting metrics, Model 3 was selected as the best-fitting model.

**Table 4:** Statistics of latent class model fit metrics for adherence to phase I cardiac rehabilitation in post-PCI patients (*N* = 212).

Model	AIC	BIC	aBIC	LMRT (*P*)	BLRT (*P*)	Entropy	Class probability
1	6,378.298	6,489.066	6,384.500	–	–	–	1
2	5,622.706	5,847.598	5,635.297	<0.001	<0.001	0.984	0.613/0.387
3	5,404.022	5,743.038	5,423.003	<0.001	<0.001	0.962	0.316/0.316/0.368
4	5,307.421	5,760.561	5,332.791	0.780	<0.001	0.974	0.179/0.326/0.332/0.163

**Figure 2:**
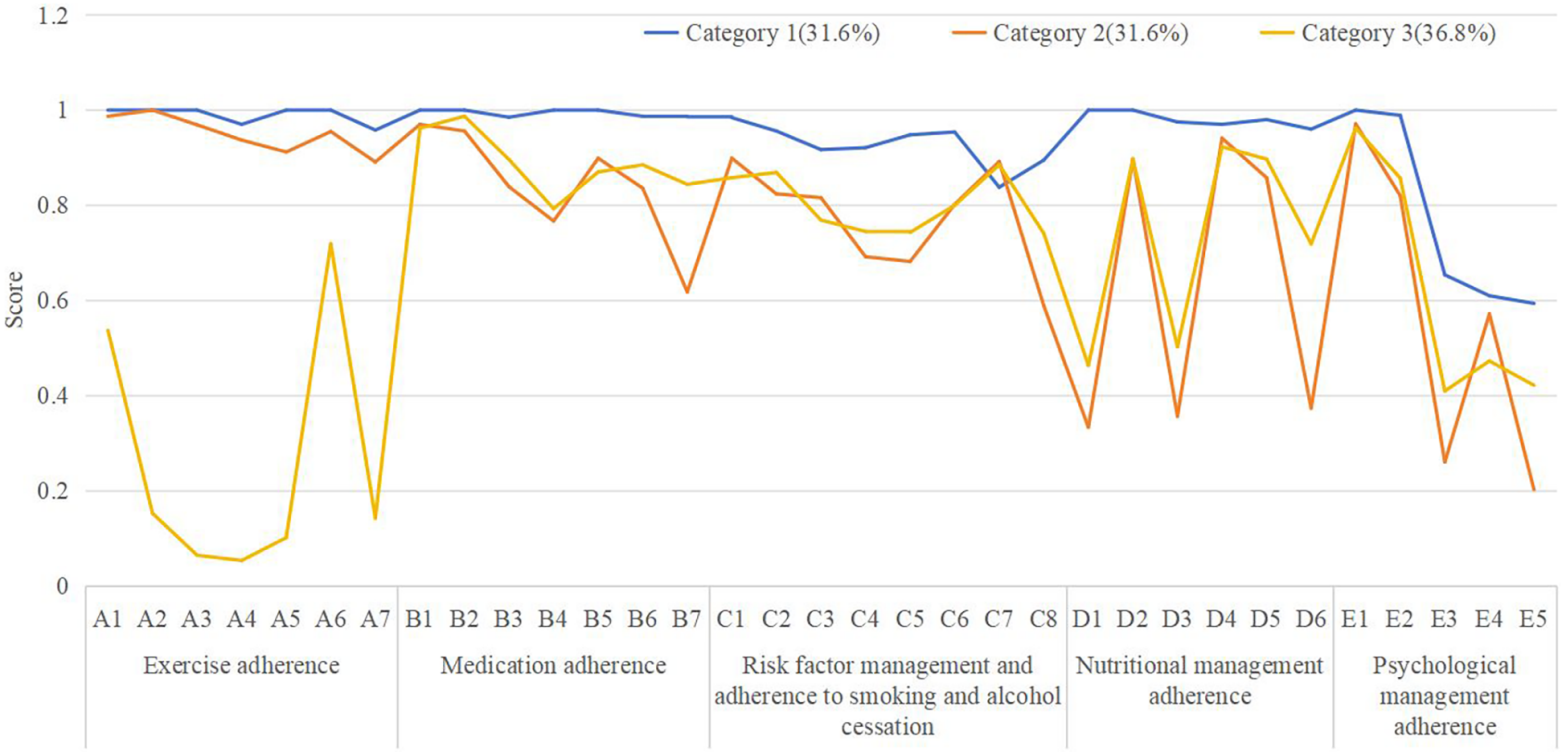
Latent class plots for the 3-category model (*N* = 212).

Latent class analysis identified three categories of adherence to Phase I cardiac rehabilitation in post-PCI patients. Category 1 had higher scores for all entries and was therefore named the **Good Adherence Group**, accounting for 31.6% (67/212). Category 2 had significantly lower nutritional adherence and psychological management adherence scores compared to the other two groups, and was named the **Poor Nutritional and Psychological Management Group**, also accounting for 31.6% (67/212). Category 3 had significantly lower exercise adherence scores and was named the **Lack of Exercise Group**, accounting for 36.8% (78/212). The distribution map is shown in Figure 2.

### 3.3 Comparison of adherence scores

The three cardiac rehabilitation adherence categories extracted by latent class analysis were categorized as the **Good Adherence Group** (31.03 ± 1.49), the **Poor Nutritional and Psychological Management Group** (24.93 ± 3.11), and the **Lack of Exercise Group** (21.81 ± 3.89). ANOVA results showed statistically significant differences in the total adherence score to Phase I cardiac rehabilitation and its subdimensions (exercise adherence, medication adherence, risk factor management, smoking and alcohol cessation adherence, nutritional management adherence, and psychological management adherence) across the three categories (*P* < 0.001 for all dimensions). The detailed ANOVA results are presented in Table 5.

**Table 5:** Comparison of adherence scores for phase I cardiac rehabilitation in different categories of post-PCI patients (*N* = 212).

Category	Number	Exercise adherence	Medication adherence	Risk factor management and adherence to smoking and alcohol cessation	Nutritional management adherence	Psychological management adherence	Total score
1	67	6.93±0.32	6.96 ± 0.27	7.42 ± 1.03	5.88 ± 0.41	3.85 ± 1.32	31.03 ± 1.49
2	67	6.66 ± 0.75	5.87 ± 1.52	6.15 ± 2.02	3.52 ± 1.21	2.73 ± 1.01	24.93 ± 3.11
3	78	1.77 ± 1.35	6.24 ± 1.34	6.38 ± 1.97	4.37 ± 1.54	3.04 ± 1.28	21.81 ± 3.89
*F* value		709.129	14.572	10.128	69.068	16.928	196.183
*P* value		<0.001	<0.001	<0.001	<0.001	<0.001	<0.001
*Post-hoc* comparison		1–3*; 2–3*	1–2*; 1–3*	1–2*; 1–3*	1–2*; 1–3*; 2–3*	1–2*; 1–3*	1–2*; 1–3*; 2–3*

1, Good adherence group; 2, Poor nutritional and psychological management group; 3, Lack of exercise group;

* *P* < 0.05.

Furthermore, *post-hoc* analyses using the LSD method were conducted to identify specific differences between groups. The results revealed that the Good Adherence Group had significantly higher adherence scores compared to the other two groups across most dimensions. The detailed *post-hoc* comparisons are also shown in Table 5.

### 3.4 Univariate analysis results

Univariate analysis showed statistically significant differences in adherence to Phase I cardiac rehabilitation among post-PCI patients with different levels of literacy, per capita monthly household income, cardiac rehabilitation knowledge scores, depression, social support, kinesiophobia, and utilization of resources for chronic diseases (*P* < 0.05). Details are shown in Table 6.

**Table 6:** Univariate analysis of potential categories of adherence to phase I cardiac rehabilitation in post-PCI patients (*N* = 212).

Item	Good adherence group (*N* = 67)	Poor nutrition and psychological management group (*N* = 67)	Lack of exercise group (*N* = 78)	*χ*^2^/F	*P*
Education level				42.873	<0.001
Primary and below	2 (3.0)	13 (19.4)	18 (23.1)		
Junior	9 (13.4)	15 (22.4)	21 (26.9)		
High school or junior college	19 (28.4)	31 (46.3)	24 (30.8)		
University and above	37 (55.2)	8 (11.9)	15 (19.2)		
Monthly per capita household income				18.297	0.006
<1,000 yuan	0	0	1 (1.3)		
1,000–3,000 yuan	0	12 (17.9)	13 (16.7)		
3,001–5,000 yuan	20 (29.9)	24 (35.8)	27 (34.6)		
>5,000 yuan	47 (70.1)	31 (46.2)	37 (47.4)		
Smoking history				10.513	0.005
No	34 (50.7)	24 (35.8)	49 (62.8)		
Yes	33 (49.3)	43 (64.2)	29 (37.2)		
Drinking history				32.729	<0.001
No	36 (53.7)	10 (14.9)	46 (59.0)		
Yes	31 (46.3)	57 (85.1)	32 (41.0)		
Cardiac rehabilitation knowledge score	40.344 ± 4.073	35.507 ± 7.811	31.321 ± 8.403	2.823	<0.001
Depressive				62.274	0.0186
Non-depressed	63 (94.1)	26 (38.8)	59 (75.6)		
Mild depression	4 (5.9)	39 (58.2)	12 (15.4)		
Moderate depression	0	2 (3.0)	4 (5.1)		
Major depression	0	0	3 (3.8)		
Social support score	44.552 ± 3.032	40.239 ± 6.349	38.474 ± 6.227	3.147	<0.001
Kinesiophobia				92.860	<0.001
Yes	10 (14.9)	10 (14.9)	14 (17.9)		
No	57 (85.1)	57 (85.1)	64 (82.1)		
Chronic disease resource utilization score	90.597 ± 10.832	82.104 ± 13.517	74.641 ± 14.143	3.654	<0.001

### 3.5 Multivariate logistic regression analysis results

Statistically significant variables from the univariate analysis were used as independent variables. The three potential categories of cardiac rehabilitation adherence (with the **Good Adherence Group** as the reference group) were used as dependent variables in a multivariate logistic regression analysis. The results are shown in Table 7.

**Table 7:** Multiple logistic regression analysis of latent classes (*N* = 212).

Latent classes	Influencing factors	*β*	Standard error	*P*	*OR* (95% CI)
Poor nutrition and psychological management group	Social support	−0.202	0.096	0.036	0.817 (0.677–0.987)
	Chronic disease resource utilization	−0.169	0.046	<0.001	0.844 (0.772–0.924)
	Education level (University and above as a reference)				
	Primary and below	3.796	2.831	0.180	44.527 (0.173–111,437.443)
	Junior	2.172	1.068	0.042	8.777 (1.082–71.226)
	High school or junior college	1.087	0.866	0.016	8.059 (1.477–43.958)
	No drinking history	−1.884	0.874	0.031	0.152 (0.027–0.842)
Lack of exercise group	Chronic disease resource utilization	−0.183	0.047	<0.001	0.833 (0.759–0.914)
	Education level (University and above as a reference)				
	Primary and below	3.357	2.752	0.222	28.704 (0.131–6,311.267)
	Junior	2.419	1.050	0.021	11.240 (1.436–88.001)
	High school or junior college	1.176	0.798	0.140	3.243 (0.679–15.488)
	No Kinesiophobia	−3.102	0.813	<0.001	0.045 (0.009–0.221)

## 4 Discussion

### 4.1 Overall findings

The results of this study showed that the total score for adherence to Phase I cardiac rehabilitation in post-PCI patients was 25.71 ± 4.77. According to the scale definition criteria, adherence to Phase I cardiac rehabilitation in post-PCI patients was at a low to moderate level. This finding is consistent with previous research that suggests post-PCI patients often face challenges in fully adhering to rehabilitation protocols (16). Using Latent Class Analysis, significant heterogeneity in adherence to Phase I cardiac rehabilitation in post-PCI patients was identified, and three categories were determined based on model adaptation results.

It is noteworthy that this study represents the first attempt to analyze adherence heterogeneity based on the five components of cardiac rehabilitation. Compared with most existing studies that focus on a single dimension (such as exercise or medication adherence) (41, 42), the innovation of this study lies in its comprehensive consideration of the impact of all five components on adherence, revealing differences in adherence across multiple dimensions among patients. This approach not only provides a more detailed perspective for cardiac rehabilitation research but also offers valuable insights for the development of personalized rehabilitation programs.

**Good Adherence Group**: 31.2% of PCI patients in this study belonged to this category, with an adherence score of 31.03 ± 1.49, demonstrating high scores across all dimensions despite the lowest percentage. *Post-hoc* analysis further confirmed that the adherence scores of this group were significantly higher across all dimensions compared to the other two groups. Research indicates that among 16,518 patients, only 13.4% adhered to current guidelines for cardiac rehabilitation, and adherence to rehabilitation has a strong independent negative correlation with mortality rates (25). Therefore, multidisciplinary efforts should focus on understanding the reasons for this group's low participation rate and promoting maximum adherence during subsequent Phase II and Phase III cardiac rehabilitation.

**Poor Nutritional and Psychological Management Group**: 32.0% of PCI patients in this study belonged to this category, with an adherence score of 24.93 ± 3.11, which was lower than the Good Adherence Group. *Post-hoc* analysis showed that nutritional and psychological management adherence were significantly lower in this group than in the Good Adherence Group. The scores for adherence to nutritional management and psychological management were 3.52 ± 1.21 and 2.73 ± 1.01, respectively. Dietary problems are an underestimated but modifiable key cardiovascular disease risk factor in current cardiac rehabilitation studies (43), and few studies have assessed patient adherence to the use of different nutritional interventions in cardiac rehabilitation. In a study of German patients with coronary artery disease, it was found that only 30% of patients followed the nutritional program prescribed during cardiac rehabilitation hospitalization (44). It is therefore urgent to prioritize improving the dietary patterns of patients after PCI. Xiao-Li Yang pointed out that the incidence of negative emotions in patients with coronary artery disease after PCI was high, and this study also confirmed that psychological management for post-PCI patients was poor (45). This suggests that medical personnel should pay greater attention to assessing the psychological situation of post-PCI patients and prevent or reduce the occurrence and development of negative emotions in these patients.

**Lack of Exercise Group**: 36.8% of PCI patients in this study belonged to this category, with an adherence score of 21.81 ± 3.89, lower than that of the other adherence categories, including a score of 1.77 ± 1.35 for exercise adherence. *Post-hoc* analysis revealed that exercise adherence scores were significantly lower in this group than in the Good Adherence Group and the Poor Nutritional and Psychological Management Group. The results of this group were lower than the percentage of completed Phase I cardiac rehabilitation exercise (41.49%) surveyed by the National Audit Office of Cardiac Rehabilitation of the United Kingdom (46), which is in line with the exercise adherence rates in Phase I of post-PCI patients in China (23.2%–46.7%) (47). This suggests that a lack of exercise is common among post-PCI patients globally. Future efforts should focus on the level of physical activity in post-PCI patients, clarify the reasons for their poor exercise adherence, and implement appropriate interventions to promote rehabilitation exercise and improve overall health status in this group.

### 4.2 Factors affecting latent class

#### 4.2.1 Social support

The results of this study showed that post-PCI patients with lower levels of social support were more likely to belong to the Poor Nutritional and Psychological Management Group. Previous studies have also confirmed a positive correlation between social support and adherence to cardiac rehabilitation (48). A lack of social support can lead to feelings of isolation and negative emotions, which in turn may result in poorer adherence to cardiac rehabilitation (49). Similar research has found that patients often require additional emotional and social support after a cardiac event (50). Therefore, healthcare professionals should engage in supportive communication with patients to understand their unique needs and collaboratively design personalized support strategies. It is crucial to recognize that such individualized support can enhance adherence and improve overall rehabilitation outcomes. From the patient's perspective, this approach allows for better provision of comprehensive psychological and social support, helping patients overcome challenges during the rehabilitation process and encouraging more active participation in their treatment and recovery.

#### 4.2.2 Chronic disease resource utilization

Patients with higher chronic disease resource utilization were more likely to belong to the Good Adherence Group. This finding is consistent with previous studies, where patients with higher resource utilization were better able to understand the content, process, and benefits of cardiac rehabilitation and showed less resistance or distrust toward it (22). Similarly, other research has shown a significant positive correlation between chronic illness resources and self-management behaviors in patients with coronary heart disease (51). However, PCI patients typically access Phase I cardiac rehabilitation resources primarily through their attending physician and the responsible nurse. Therefore, clinical multidisciplinary rehabilitation teams should establish a comprehensive patient education platform, where patients can access the latest rehabilitation information, treatment plans, nutritional advice, and more. This platform should facilitate self-learning and real-time communication with medical staff, ensuring the timely updating and interaction of information. Such an approach can enhance patients' recognition and adherence to cardiac rehabilitation, leading to improved rehabilitation outcomes.

#### 4.2.3 Education level

PCI patients with a middle school or high school/junior college education were more likely to belong to the Poor Nutritional and Psychological Management Group. Meanwhile, PCI patients with middle school education were more likely to belong to the Lack of Exercise Group. Patients with a higher level of education were better able to understand and apply the acquired knowledge and resources to cardiac rehabilitation, and to recognize changes in their condition in time to seek professional support (52). Patients with lower education levels often struggle to acquire and understand professional health information, lacking a correct perception of cardiac rehabilitation, which results in greater difficulties in their cardiac rehabilitation treatment (53). Based on the patient's education level, clinicians should provide tailored educational materials and implement comprehensive interventions to address common psychological barriers, such as fear or anxiety about the rehabilitation process. By combining educational support with psychological interventions, patients can be helped to overcome these psychological challenges.

#### 4.2.4 Drinking history

This study found that PCI patients with a history of alcohol consumption were more likely to belong to the Poor Nutritional and Psychological Management Group. Alcohol consumption is often associated with high-fat, high-salt dietary habits (54), which suggests that when designing nutritional prescriptions for cardiac rehabilitation, the patient's daily lifestyle habits should be taken into consideration. Several studies have also shown that alcohol consumption not only affects dietary habits but is also closely linked to psychological well-being, which may exacerbate difficulties during cardiac rehabilitation (55). In future clinical practice, PCI patients with a history of alcohol consumption should receive regular alcohol use assessments, along with a personalized alcohol cessation plan and progress tracking system. Special attention should also be given to their diet and psychological well-being. The medical team should guide proper food choices and emphasize the long-term benefits of lifestyle changes, such as quitting alcohol, eating a balanced diet, and maintaining a positive mindset. These comprehensive interventions can improve recovery outcomes and enhance the long-term prognosis of cardiac health.

#### 4.2.5 Kinesiophobia

Research has found that individuals with Kinesiophobia are more likely to belong to the group that lacks physical activity. Bäck similarly confirmed that patients with Kinesiophobia are unlikely to participate in exercise-based cardiac rehabilitation (56). Previous qualitative studies have indicated that Kinesiophobia in patients may lead to negative beliefs and attitudes toward physical activity, as well as a lack of support and information from healthcare providers (57). Therefore, healthcare professionals should first assess the root causes of a patient's Kinesiophobia and address concerns like physical discomfort or heart strain during activity. By building trust, a personalized exercise plan can be developed, starting with low-intensity activities and gradually increasing intensity. Providing timely feedback and support will help build the patient's confidence. These interventions can help overcome Kinesiophobia, improving both physical health and quality of life, and promoting long-term cardiovascular health.

## 5 Limitations and future directions

This study utilized a self-reported questionnaire for data collection. Although strict quality control measures were implemented, reporting bias cannot be completely eliminated. Moreover, the sample used in this study was relatively homogeneous, and the sample size was limited. Therefore, future research could benefit from multi-center sampling to gain a more comprehensive understanding of the characteristics of different types of PCI patients.

It is worth noting that all participants in this study were undergoing PCI for the first time without a history of coronary revascularization. This characteristic may affect the generalizability of the study findings. Since all participants had no prior experience with cardiac rehabilitation, the lack of previous rehabilitation experience may further limit the external validity of the results.

This study did not provide detailed information about the specific PCI procedures or clinical characteristics of the patients, especially regarding comorbidities such as heart failure and renal failure. The absence of such data could limit the external validity and generalizability of the findings. Although Latent Class Analysis (LCA) revealed adherence patterns, the exclusion of these clinical characteristics might limit the ability to further refine the adherence differences across patient groups. Therefore, future research should consider including these clinical factors (such as comorbidities) in the analysis to identify more refined patient clusters and further explore how these factors influence adherence to cardiac rehabilitation.

Moreover, longitudinal study designs could facilitate the exploration of the dynamic changes in cardiac rehabilitation adherence after PCI, providing a deeper understanding of the formation and development of post-PCI adherence.

## 6 Conclusion

This study found that adherence to Phase I cardiac rehabilitation among post-PCI patients in China remains moderate to low. Latent class analysis confirmed that post-PCI patients' adherence to Phase I cardiac rehabilitation exhibits distinct categorical patterns, including a good adherence group, a poor nutrition and psychological management group, and a lack of exercise group. Factors influencing these different latent subgroups include limited social support, poor utilization of chronic disease resources, low education levels, a history of alcohol consumption, and the presence of Kinesiophobia. This suggests that healthcare providers should accurately identify the adherence categories of post-PCI patients in Phase I cardiac rehabilitation and implement targeted interventions to improve their in-hospital rehabilitation adherence, thereby promoting better health outcomes for post-PCI patients.

## Data Availability

The original contributions presented in the study are included in the article/Supplementary Material, further inquiries can be directed to the corresponding authors.
