# Supplementary material for: Adherence to phase I cardiac rehabilitation in post-PCI patients: a latent class analysis
Source: Front Cardiovasc Med. 2025 Feb 24;12:1460855. doi: 10.3389/fcvm.2025.1460855 (PMC11891241; doi:10.3389/fcvm.2025.1460855)
Supplement: Supplementary file 1 [file Table1.pdf]

Supplementary Table 1 The Cardiac Rehabilitation Adherence Evaluation Scale for Coronary Heart Disease Patients

Exercise adherence		
A1. Do you follow the doctor's instructions to participate in rehabilitation exercises (choose one or more, such as walking, swimming, cycling, tai chi, aerobics, strength training, yoga, climbing stairs, etc.)?	A. Yes	B. No
A2. Do you exercise the required number of times per week as instructed by your doctor? (≥ 3 times/week)	A. Yes	B. No
A3. Do you meet the duration requirements for each exercise session as instructed by your doctor? (30 minutes or more)	A. Yes	B. No
A4. Do you follow the doctor's instructions to perform warm-up and cool-down exercises?	A. Yes	B. No
A5. Do you persistently exercise without stopping, as advised by your healthcare provider?	A. Yes	B. No
A6. If you experience discomfort during exercise, do you stop immediately?	A. Yes	B. No
A7. Do you understand the importance of exercise for disease treatment?	A. Yes	B. No
Medication adherence		
B1. Do you always take your medications as prescribed?	A. Yes	B. No
B2. Do you always purchase your medications as prescribed?	A. Yes	B. No
B3. When your condition changes, do you adjust your medication on your own?	A. Yes	B. No
B4. Do you sometimes forget to take your medication?	A. Yes	B. No
B5. Do you experience difficulties taking your medication?	A. Yes	B. No
B6. Do you understand the effects, side effects, and precautions of your medications?	A. Yes	B. No
B7. Do you understand the importance of medication for disease treatment?	A. Yes	B. No
Risk factor management and adherence to smoking and alcohol cessation		
C1. Do you follow the advice of healthcare professionals to control your	A. Yes	B. No

blood pressure, blood sugar, and blood lipids?

C2. Do you follow the healthcare provider's advice to keep your weight within a normal range? A. Yes B. No

C3. Can you refrain from smoking as advised by your healthcare provider? A. Yes B. No

C4. Can you actively avoid environments with smoking to avoid secondhand smoke, as advised by your healthcare provider? A. Yes B. No

C5. Do you follow your healthcare provider's advice to limit or stop drinking alcohol? A. Yes B. No

C6. Do you follow your healthcare provider's advice to reduce salt, fat, and sugar intake? A. Yes B. No

C7. Do you follow your healthcare provider's advice to attend follow-up visits as scheduled? A. Yes B. No

C8. Do you understand the importance of controlling risk factors (e.g., blood pressure, blood sugar, and lipids) and regular follow-ups for disease treatment? A. Yes B. No

Nutritional management adherence

D1. Do you consume less than 6 grams of salt per day (about one beer bottle cap or less)? A. Yes B. No

D2. Do you consume less than 30 grams of oil per day (about one-third of a standard oil spoon)? A. Yes B. No

D3. Do you pay attention to your diet by eating fewer processed foods, fatty meats, animal skin, organ meats, sausages, etc.? A. Yes B. No

D4. Do you increase your intake of fresh vegetables, fruits, and nuts? A. Yes B. No

D5. Do you make sure to eat regular meals every day without overeating? A. Yes B. No

D6. Do you understand the importance of diet for disease treatment? A. Yes B. No

Psychological management adherence

E1. Do you believe in your ability to overcome your disease? A. Yes B. No

E2. When you feel negative emotions, can you adjust your mood using various methods? (e.g., listening to music, relaxing, deep breathing, etc.)	A. Yes B. No
E3. When encountering troubling or irritating situations, can you control your emotions and handle them calmly and rationally?	A. Yes B. No
E4. Do you ensure you get enough rest and maintain sufficient sleep?	A. Yes B. No
E5. Do you understand the importance of psychological management and sleep for disease treatment?	A. Yes B. No
