# Supplementary material for: Adherence to phase I cardiac rehabilitation in post-PCI patients: a latent class analysis
Source: Front Cardiovasc Med. 2025 Feb 24;12:1460855. doi: 10.3389/fcvm.2025.1460855 (PMC11891241; doi:10.3389/fcvm.2025.1460855)
Supplement: Supplementary file 2 [file Datasheet1.docx]

**General information questionnaire**

Guideline: Please read the following questions carefully, and according to your own situation, please fill in your real information in the corresponding horizontal line and put "√" in the corresponding position.

Name： Time：

| **Sex：**①Male□ ②Female□ | **Age：** |
| --- | --- |
| **Residence：**①Living alone□ ②Not living alone□ | |
| **Marital status：**①Married□ ②Other□ | |
| **Education level：**①Primary and below□ ②Junior□ ③High school or junior college□ ④University and above□ | |
| **Methods of payment of medical expenses：**①Self-financed□ ②Medical insurance□ | |
| **Monthly per capita household income：**①＜1000yuan□ ②1000-3000yuan□  ③3000-5000yuan□ ④＞5000yuan□ | |
| **Past histories：**①hypertensive□ ②hyperlipidemia□ ③diabetes□  ④stroke□ ⑤other□ ⑥no□ | |
| **Smoking history：**①no□ ②yes□  **Drinking history：**①no□ ②yes□ | |
| **Contact details (telephone)：** | |

**Cardiac Rehabilitation Adherence Questionnaire**

Guideline: Below is a list of possible situations that may arise during your cardiac rehabilitation, please read each question carefully and select the answer that matches your situation by ticking the appropriate letter at the end of the question.

| 一、exercise adherence | | |
| --- | --- | --- |
| A1.Did you participate in exercise training as required by your doctor during or after your hospitalisation? (Yes to one or more of these, No to none.) For example, walking, running, swimming, dancing, gymnastics, Tai Chi, acupressure point tapping, bedside exercise training, stationary bike, push-ups, dumbbells, muscle and ligament stretching, etc.) | A.yes | B.no |
| A2.Do you train for exercise the number of days per week recommended by your doctor? (≥3 days/week) | A.yes | B.no |
| A3.Does the amount of time you spend exercising each day meet the requirements of the exercise programme set by your doctor? (30min/time) | A.yes | B.no |
| A4.Do you train for exercise at the intensity prescribed by your doctor? | A.yes | B.no |
| A5.Do you follow your healthcare provider's advice to keep exercising without interruption? | A.yes | B.no |
| A6.When you experience discomfort during exercise, do you stop exercising immediately? | A.yes | B.no |
| A7.Do you understand the importance of sensible exercise in the treatment of disease? | A.yes | B.no |
| 二、medication adherence | | |
| B1.Do you always take your medication on time as prescribed? | A.yes | B.no |
| B2.Do you always follow your doctor's instructions and take your medication in the right amount? | A.yes | B.no |
| B3.Do you adjust your medication on your own when your condition changes? | A.yes | B.no |
| B4.Do you sometimes forget to take your medication? | A.yes | B.no |
| B5.Do you find it difficult to adhere to your medication? | A.yes | B.no |
| B6.Do you understand the effects of medications, their side effects and their precautions? | A.yes | B.no |
| B7.Do you understand the importance of following medical advice and taking medication appropriately for the treatment of your illness? | A.yes | B.no |
| 三、Risk factor management and adherence to smoking and alcohol cessation | | |
| C1.Are you able to control your blood pressure, blood glucose and blood lipids in accordance with the advice of your healthcare provider? | A.yes | B.no |
| C2.Are you able to keep your weight within the normal range by following your healthcare provider's instructions? | A.yes | B.no |
| C3.Are you able to keep your waistline within reasonable limits by following your healthcare provider's teachings? | A.yes | B.no |
| C4.Are you able to comply with healthcare professionals' advice not to smoke? | A.yes | B.no |
| C5.Do you avoid secondhand smoke by staying away from smoking environments as instructed by your healthcare provider? | A.yes | B.no |
| C6.Are you able to follow your healthcare provider's advice not to drink alcohol or to limit your alcohol consumption to a lesser extent? | A.yes | B.no |
| C7.Do you follow your healthcare provider's instructions for regular review? | A.yes | B.no |
| C8.Do you understand the importance of smoking cessation and alcohol restriction, controlling risk factors such as blood pressure, blood glucose, blood lipids, weight and regular check-ups in the treatment of your disease? | A.yes | B.no |
| 四、Nutritional management adherence | | |
| D1.Do you consume <6 grams of salt per day (about one cap of a beer bottle)? (<3 grams for heart failure patients) | A.yes | B.no |
| D2.Do you eat ≤ 30g of cooking oil per day (about three white porcelain spoons) | A.yes | B.no |
| D3.Do you watch your diet and eat less or no fried, pickled, carbon smoked foods, fatty meats, meat skins, sausages, ham, butter, cream, etc.? | A.yes | B.no |
| D4.Are you increasing the intake of fresh vegetables, fruits and nuts in your diet? | A.yes | B.no |
| D5.Do you manage to chew slowly and not overfill each meal? | A.yes | B.no |
| D6.Do you understand the importance of proper diet in the treatment of diseases? | A.yes | B.no |
| 五、Psychological management adherence | | |
| E1.Are you able to correctly understand your disease and have the confidence to overcome it? | A.yes | B.no |
| E2.When you are feeling down, are you able to regulate your emotions in a variety of ways? (e.g. listening to music, talking, deep breathing, meditation, etc.) | A.yes | B.no |
| E3.When faced with an emergency or an irritating situation, are you able to keep your emotions at bay and calmly search for an appropriate solution? | A.yes | B.no |
| E4.Are you able to stay up late and get enough sleep? | A.yes | B.no |
| E5.Are you aware of the importance of proper mental management and sleep in the treatment of your illness? | A.yes | B.no |

**Cardiac Rehabilitation Knowledge Questionnaire**

Guideline: Please read the following questions carefully, and according to your own knowledge of cardiac rehabilitation, please select and tick the appropriate place

| 1.Cardiac Rehabilitation Basics: | | |
| --- | --- | --- |
| （1）Do you know what cardiac rehabilitation involves? | know | Don't know |
| （2）Cardiac rehabilitation can control the various risk factors for cardiovascular disease and slow down the disease process. | know | Don't know |
| （3）Cardiac rehabilitation improves exercise tolerance, lowers blood lipid levels, and reduces the rate of restenosis in the coronary arteries. | know | Don't know |
| 2.Exercise workouts for cardiac rehabilitation: | | |
| （1）Purpose of sports and exercise | know | Don't know |
| （2）The Importance of Sports and Exercise | know | Don't know |
| （3）Methods of Exercise Exercise | know | Don't know |
| 3.Risk factor control: | | |
| （1）Regular exercise can control coronary heart disease risk factors | know | Don't know |
| （2）Evidence-based medication can control risk factors | know | Don't know |
| （3）Healthy living diet can control risk factors | know | Don't know |
| （4）Regular check-ups and receiving health guidance | know | Don't know |

| 4.Health Liability Component: | | | |
| --- | --- | --- | --- |
| （1）Blood pressure should be tightly controlled | very necessary | necessary | Don't necessary |
| （2）Diabetes should be treated aggressively | very necessary | necessary | Don't necessary |
| （3）You should eat a low-fat diet. | very necessary | necessary | Don't necessary |
| （4）Should be on a low-salt diet (<6g of salt per day) | very necessary | necessary | Don't necessary |
| （5）You should be careful about losing weight and controlling it | very necessary | necessary | Don't necessary |
| （6）You should stop smoking and limit alcohol. | very necessary | necessary | Don't necessary |
| （7）Lipid and glucose levels should be monitored regularly after discharge from hospital | very necessary | necessary | Don't necessary |
| （8）You should always learn about cardiac rehabilitation and exercise workouts | very necessary | necessary | Don't necessary |
| （9）Post-operative adherence to exercise is as important as treatment | very necessary | necessary | Don't necessary |
| （10）Exercise should be done daily (walks, housework) | very necessary | necessary | Don't necessary |
| （11）Regular outpatient check-ups should be done | very necessary | necessary | Don't necessary |

**PHQ-9**

| question | A No or little time | B slice | C a fair amount of time | D Most or all of the time |
| --- | --- | --- | --- | --- |
| 1.Not interested in what's going on. |  |  |  |  |
| 2.Feeling low, depressed and hopeless |  |  |  |  |
| 3.Inability to fall asleep or sleeping too long |  |  |  |  |
| 4.Feeling tired or lacking energy |  |  |  |  |
| 5.No appetite or binge eating |  |  |  |  |
| 6.Feeling guilty about yourself or feeling like a YES loser or causing your family to be unsuccessful |  |  |  |  |
| 7.Inability to concentrate on things: such as reading the newspaper or a book |  |  |  |  |
| 8.Walking or talking rather slowly or with unusual excitement and walking around |  |  |  |  |
| 9.Thinking it's better to die or self-harm. |  |  |  |  |
| totals |  |  |  |  |

**Social Support Rating Scale**

Guideline: Please read the following questions carefully and, depending on your situation, please select and tick the appropriate position

| **1．How many friends do you have who are close enough to get support and help? (choose only one)**  (1)Not a single one. (2)1～2 (3)3～5 (4)6 or more  **2．In the last year you: (check one only)**  (1)Away from family and alone in a room.  (2)Residence changes frequently, mostly with strangers.  (3)Living with classmates, colleagues or friends (4)Living with family. |
| --- |
| **3．You and your neighbours: (check one only)**  **4．You and a colleague: (check one only)**  **Shared Options：**  (1)Never cared for each other, only nodded their heads.  (2)Might be slightly concerned when in trouble.  (3)Some neighbours/colleagues are concerned about you.  (4)Most neighbours/colleagues care about you. |
| **5．Support and care received from family members (tick the appropriate box)**   \|  \| no \| very little \| usual \| full support \| \| --- \| --- \| --- \| --- \| --- \| \| Husband and wife (lovers) \|  \|  \|  \|  \| \| sons and daughters \|  \|  \|  \|  \| \| siblings \|  \|  \|  \|  \| \| Other members (e.g. sister-in-law) \|  \|  \|  \|  \| |
| **6．In the past, the sources of financial support and help in solving practical problems that you have received in times of emergency are:**  **7．In the past, the sources of comfort and concern you used to receive when you were in an emergency situation were:**  (1)No source。  (2)The following sources: (check all that apply)  A．mate B．Other family members C．friends D．a relative (i.e. family relation) E．co-worker F．work unit G．Official or semi-official organisations such as party groups and trade unions H．Non-official organisations such as religious and social groups I．other than |
| **8．How you talk about your troubles: (check one only)**  (1)Never tell anyone about it.  (2)Speak only to one or two people with whom you have a very close relationship.  (3)You will speak up if a friend initiates the enquiry.  (4)Take the initiative to talk about your troubles in order to gain support and understanding.  **9．How do you seek help when you are troubled: (check one only)**  (1)Rely only on yourself and accept no help from others.  (2)Rarely asks for help.  (3)Sometimes asking for help.  (4)Often seek help from family, friends, relatives and organisations when in difficulty. |
| **10．For organising activities by groups (e.g. party groups, religious organisations, trade unions, student unions, etc.), do you: (check one only)**  (1)never attended (2)occasional  (3)regular participation (4)Active participation and positive activities. |

**The Tampa Scale for Kinesiophobia Heart (TSK-SV Heart)**

Guideline: Please read the following entries carefully, the following questions are used to reflect your fear of exercise in your daily life, please select and tick the corresponding positions

| question | Completely disagree. | disagree | agree | wholeheartedly agree |
| --- | --- | --- | --- | --- |
| 1.I'm worried about getting hurt during the activity/exercise. |  |  |  |  |
| 2.If I try to be active/exercise, my heart problems worsen. |  |  |  |  |
| 3.I think I have a serious health problem at the moment. |  |  |  |  |
| 4.Activity/exercise may have given me relief from my heart problems. |  |  |  |  |
| 5.My family/friends feel unappreciative of my physical condition. |  |  |  |  |
| 6.Later in life, heart disease would cause me to be weak. |  |  |  |  |
| 7.Physical injuries usually trigger heart problems. |  |  |  |  |
| 8.Some things cause discomfort in my chest, they're not necessarily dangerous. |  |  |  |  |
| 9.I'm afraid I'll hurt myself by accident. |  |  |  |  |
| 10.By avoiding some unnecessary activities, I can prevent my heart problems from getting worse. |  |  |  |  |
| 11.As long as I'm not doing anything physically harmful, it won't cause heart problems. |  |  |  |  |
| 12.Even though I have heart problems, regular activity/exercise would control it better . |  |  |  |  |
| 13.Heart problems suggest when I should stop the activity so I don't hurt myself. |  |  |  |  |
| 14.It is not safe for someone in my condition to be active/exercise. |  |  |  |  |
| 15.I can't do things like everyone else because of the risk of heart problems. |  |  |  |  |
| 16.Some activities/exercises can create heart problems, but I don't think they are dangerous. |  |  |  |  |
| 17.When a person has a heart problem, he/she should not be active/exercising. |  |  |  |  |

**Chronic Disease Resource Survey Questionnaire**

Guideline: Please read the following entries carefully and, based on the real situation in the past month, tick the box after each entry to select the one that suits you best.

| question | never | infrequent | medium level | more | much |
| --- | --- | --- | --- | --- | --- |
| 1．Do doctors and nurses discuss things related to cardiac rehabilitation with you |  |  |  |  |  |
| 2．Did the doctor or nurse listen patiently to your description of your illness? |  |  |  |  |  |
| 3．Did the doctor or nurse carefully explain your lab results? |  |  |  |  |  |
| 4．Does a family member or friend exercise and workout with you? |  |  |  |  |  |
| 5．Do you share diet recipes with family or friends? |  |  |  |  |  |
| 6．Does a family member or friend buy or prepare a healthy diet for you that is conducive to managing your condition? |  |  |  |  |  |
| 7．Are you focusing on the things you can do well in cardiac rehab and not dwelling on the things you can't do? |  |  |  |  |  |
| 8．Have you thought about how to achieve your cardiac rehabilitation goals (e.g., diet, smoking cessation control, etc.)? |  |  |  |  |  |
| 9．Do you organise things so as to facilitate your heart recovery? |  |  |  |  |  |
| 10．Do you walk around your home or do outdoor sports for exercise? |  |  |  |  |  |
| 11．Do you walk or do exercise with your neighbours? |  |  |  |  |  |
| 12．Do you eat at restaurants that offer a wide variety of tasty and low-salt foods? |  |  |  |  |  |
| 13．Do you go to the park for walks, picnics or other outdoor activities (neighbourhood fitness area, chess, etc.)? |  |  |  |  |  |
| 14．Do you have health insurance that reimburses cardiac rehab and to what extent? |  |  |  |  |  |
| 15．Have you seen any adverts or campaigns encouraging people not to smoke, to eat a low-salt, low-fat diet, and to get regular exercise? |  |  |  |  |  |
| 16．Have you watched any health-related programmes or articles (e.g. through cardiac rehabilitation educational brochures, television, microblogging, mobile phone websites, etc.) |  |  |  |  |  |
| 17．Have you ever attended a free or low-cost seminar in a hospital, community or other organisation that would benefit you in managing your illness? |  |  |  |  |  |
| 18．Do you participate in any local organised activities such as chess clubs, calligraphy classes etc. for hobbies or other reasons? |  |  |  |  |  |
| 19．Do you participate in an exercise team or use fitness equipment provided by the neighbourhood? (e.g. neighbourhood square dancing, morning jogging, cycling teams, etc.) |  |  |  |  |  |
| 20．Do you have more flexible working hours that can be adapted to meet your needs? |  |  |  |  |  |
| 21．Does your workplace have rules that make it easier for you to manage illness (e.g. no smoking, morning exercises, etc.)? |  |  |  |  |  |
| 22．Can you co-ordinate things at your work when you make decisions or choices? |  |  |  |  |  |
